# 

**Published:** 1900-03-24

**Authors:** 


THE HOSPITAL. "The standard of highest purity."—*Lancet.* MARCH 24TH, 1900.

**CADBURY'S COCOA** ABSOLUTELY PURE.

**CADBURY'S COCOA** NO DRUGS OR ALKALIES USED.

### THE HOSPITAL

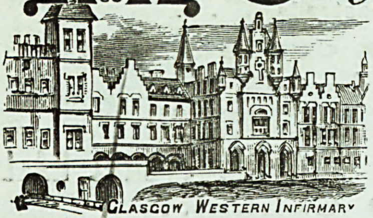

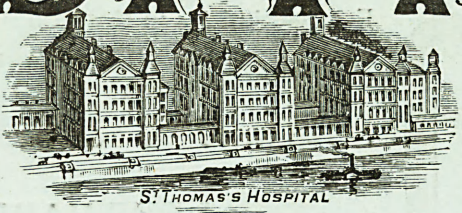

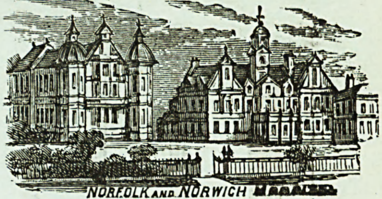

No. 704. Vol. XXVII. [Registered as a Newspaper.] SATURDAY, MARCH 24, 1900. [Subscription Terms, see p. 410.] **PRICE TWOPENCE.**

